# Glycocalyx in Atherosclerosis-Relevant Endothelium Function and as a Therapeutic Target

**DOI:** 10.1007/s11883-017-0691-9

**Published:** 2017-11-10

**Authors:** Ronodeep Mitra, Gerard Leland O’Neil, Ian Chandler Harding, Ming Jie Cheng, Solomon Arko Mensah, Eno Essien Ebong

**Affiliations:** 1 Department of Bioengineering, Northeastern University, Boston, MA USA; 2 Department of Biology, Northeastern University, Boston, MA USA; 3 Department of Chemical Engineering, Northeastern University, 360 Huntington Avenue 313 Snell Engineering Building, Boston, MA 02115 USA; 4 Department of Neuroscience, Albert Einstein College of Medicine, New York, NY USA

**Keywords:** Atherosclerosis, Endothelial dysfunction, Endothelial glycocalyx, Cardiovascular disease treatment

## Abstract

**Purpose of Review:**

The cell surface-attached extracellular glycocalyx (GCX) layer is a major contributor to endothelial cell (EC) function and EC-dependent vascular health and is a first line of defense against vascular diseases including atherosclerosis. Here, we highlight our findings regarding three GCX-dependent EC functions, which are altered when GCX is shed and in atherosclerosis. We discuss why the GCX is a viable option for the prevention and treatment of atherosclerosis.

**Recent Findings:**

GCX regulated EC activities such as barrier and filtration function, active cell-to-cell communication, and vascular tone mediation contribute to function of the entire vascular wall. Atheroprone vessel regions, including bifurcation sites, exhibit breakdown in GCX. This GCX degradation allows increased lipid flux and thereby promotes lipid deposition in the vessel walls, a hallmark of atherosclerosis. GCX degradation also alters EC-to-EC communication while increasing EC-to-inflammatory cell interactions that enable inflammatory cells to migrate into the vessel wall. Inflammatory macrophages and foam cells, to be specific, appear in early stages of atherosclerosis. Furthermore, GCX degradation deregulates vascular tone, by causing ECs to reduce their expression of endothelial nitric oxide synthase (eNOS) which produces the vasodilator, nitric oxide. Loss of vasodilation supports vasoconstriction, which promotes the progression of atherosclerosis.

**Summary:**

Common medicinal atherosclerosis therapies include lipid lowering and anti-platelet therapies. None of these treatments specifically target the endothelial GCX, although the GCX is at the front-line in atherosclerosis combat. This review demonstrates the viability of targeting the GCX therapeutically, to support proper EC functionality and prevent and/or treat atherosclerosis.

## Introduction

Cardiovascular disease (CVD), the leading cause of death worldwide, affects 92 million adults in the United States (US) [1]. Over 600,000 of these people die annually from subsequent heart disease, accounting for 25% of US deaths [1]. When separately considered from other CVDs, stroke is 5th among all causes of death in the US, killing nearly 133,000 people per year [1]. The precursor to most CVDs is atherosclerosis, which occurs because of dysfunction of the vasculoprotective endothelial cell (EC) lining of the inner blood vessel wall [2]. Both atherogenesis and EC dysfunction have been noted to coincide with the loss of the cell surface-attached glycocalyx (GCX) that coats ECs [2–5]. Therefore, a potential approach to restoring normal EC functionality to prevent or treat atherosclerosis is to target and regenerate the GCX layer in compromised areas of blood vessel walls.

The primary aim of this review is to highlight the role of the GCX as a contributor to three key EC functions relevant to vascular health and atherosclerosis. Additionally, we will evaluate its potential as a target for therapies that treat atherosclerosis.

## Atherosclerosis Etiology

Atherosclerosis is a chronic arterial vessel disease, characterized by the accumulation of plaque and subsequent erosion or rupture of some “vulnerable” or “high risk” plaques [6]. The disease is preferentially located in regions of the vasculature where blood flow is disturbed by geometric complexity such as bends or branch points [7]. The aortic arch curvature and carotid sinus bifurcation, for example, cause blood flow disruptions and irregularities that can be characterized by recirculating flows and steep spatial variation in the magnitude and direction of wall shear stress [8]. Atherosclerosis generally does not form in straight structures of the vasculature such as the descending thoracic aorta, where blood flow is more uniform and unidirectional [8, 9].

Dysfunction of the flow sensitive vasculoprotective endothelium is a first step in the atherosclerosis process [2]. The endothelium is the innermost cell layer of the arterial wall and, therefore, lies at the crucial interface between the blood and vascular tissue compartments [2]. Endothelium dysfunction permits low-density lipoprotein (LDL) and its apolipoprotein B to leave the blood compartment and accumulate in the subendothelial space [7]. The retained lipoproteins are susceptible to modification by oxidation, enzymatic cleavage, and aggregation [10, 11], all of which stimulate inflammation [12]. The inflammatory response includes the recruitment of monocytes, which transmigrate across the endothelial monolayer into the intima, where they proliferate and differentiate into macrophages [13]. The macrophages then uptake the lipoproteins, developing into foam cells [14]. Lesions continue to expand due to the migration of new mononuclear cells, which are then accompanied by cell proliferation and further accumulation of extracellular lipids [13–15]. A change in the nature of extracellular matrix production also occurs [13–15], characterized by a switch from mostly elastin (and consequent reduction in vessel wall elasticity) to collagen (causing vessel wall hardening). Eventually, atherosclerotic lesions undergo the formation of an overlying scar, called the fibrous cap [16, 17]. The fibrous cap, which is in constant dynamic equilibrium, provides a protective barrier between platelets in the blood stream and pro-thrombotic plaque contents [15].

An increase in plaque size can cause narrowing of the vessel lumen or complete obstruction of blood flow, causing hypoxic conditions to specific organs in the body like the brain or heart. Alternatively, large plaques “hidden” in the vessel wall in regions of outward remodeling can disrupt on the luminal surface to form a thrombus [18]. This is common for advanced plaques in which foam cells die and contribute their lipid-filled contents to destabilizing necrotic cavities within the plaques [13] while matrix degradation enzymes expand the cavities to create large voids. Following plaque destabilization and subsequent rupture, pro-thrombotic material on the plaque remnant surface is exposed to flowing blood. Local occlusion of the pro-thrombotic plaque remnant, primarily by recruitment and adhesion of circulating platelets, can cause obstruction of blood flow. Additionally, embolism can occur when ruptured plaque fragments travel to and block distant blood vessels. Rupture of atherosclerotic plaques is the most common cause of strokes and myocardial infarctions [2].

## The Endothelium and its Protective Glycocalyx

Because the condition of the vascular endothelium is a major contributor to the balance between vascular health and the progression of atherosclerosis [2], understanding EC function has been the focus of intense research for many decades [19, 20]. Vascular ECs line the entire circulatory system and were once thought to be inactive [21] but are now known to have very distinct and unique activities that are essential to vascular biology [22, 23]. Vascular ECs are directly exposed to and able to sense changes in hemodynamic forces and biochemistry of flowing blood [24, 25]. In turn, ECs respond genetically, morphologically, and functionally [12, 26, 27] to mediate modification of vasomotion, homeostasis, angiogenesis, and vascular growth [24, 25].

A well-known endothelium response to the flow environment involves synthesis and release of various vasoactive substances, including the predominant vasodilator nitric oxide [28]. ECs subjected to uniform and unidirectional flows constantly release nitric oxide [29, 30]. Nitric oxide has several anti-atherosclerotic effects in the cardiovascular system, such as inhibition of platelet aggregation, prohibition of excessive smooth muscle cell proliferation, prevention of leukocyte adhesion, and vessel dilation capabilities [31]. The endothelium also releases the vasodilators prostacyclin and endothelium derived hyperpolarizing factor (EDHF) [20]. In general, EDHF-mediated responses include an increase in the intracellular calcium concentration. This results in an endothelium-dependent hyperpolarization of smooth muscle cells, which evokes electrical coupling through myoendothelial junctions and accumulation of potassium ions in the intercellular space [32]. Additionally, the endothelium releases vasoconstrictive factors such as thromboxane and endothelin-1 [20].

Another well-characterized EC response to healthy blood flow is the tightening of junctional interconnections between neighboring ECs in order to (i) reinforce the barrier between the blood and the underlying tissue [32, 33] and (ii) link neighboring ECs so that they can communicate (Fig. 1). Barrier function keeps unwanted molecules and cells from entering and accumulating in vessel walls. EC-to-EC communication is important for the inter-cytoplasmic exchange of ions, metabolites, and other small molecules (< 1 kDa) [34, 35] that mediate many vasculoprotective EC functions [36–38].

**Figure:**
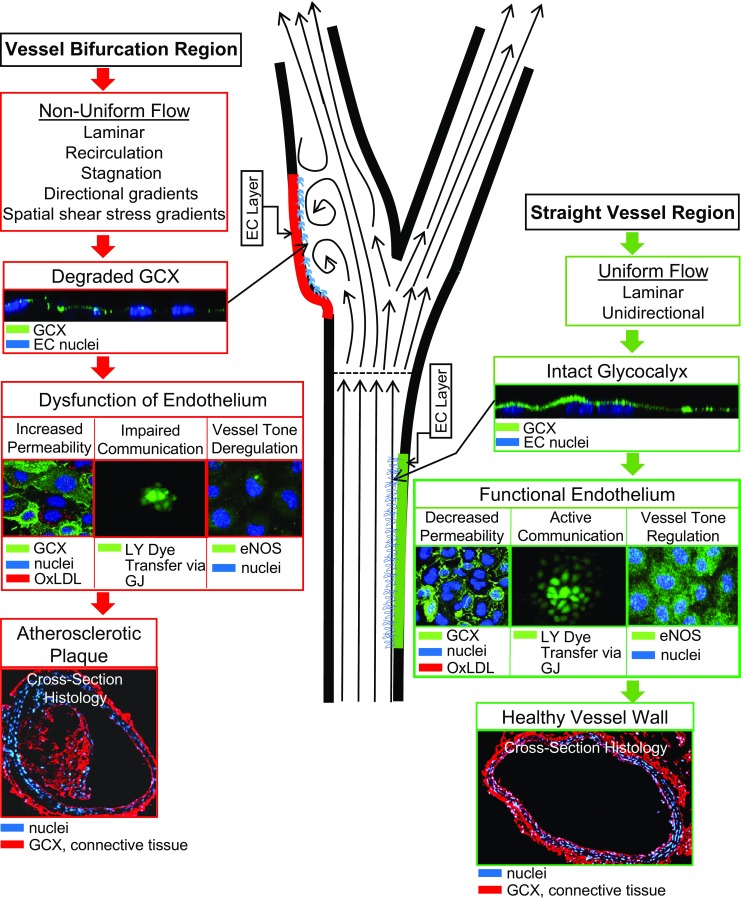

Endothelial cells become dysfunctional in disturbed blood flow conditions, particularly blood flow conditions that recirculate with high shear stress gradients as described above. This endothelial dysfunction results in impairment of nitric oxide production, re-distribution of inter-EC junctions, altered communication, and loss of barrier function [39] (Fig. 1). Thus, disturbed flow provides predisposition for atherogenic tendencies by inducing EC dysfunction whereas uniform flow shields against atherosclerosis by enhancing endothelium integrity [40–42].

**Fig. 2:**
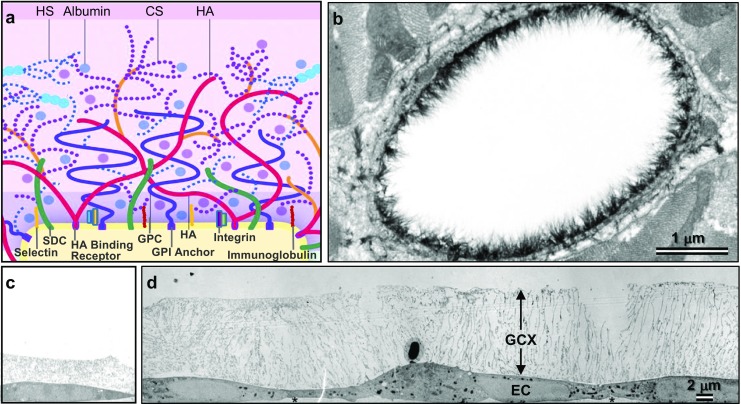
The protective GCX of the endothelium is shown here. **a** This drawing (adapted from [57]) shows selected components of the EC GCX, which include the integrins, selectins, and immunoglobulins. Syndecan and glypican are also shown. They are bound to chondroitin sulfate and heparan sulfate GAGs. Another GAG, hyaluronic acid is also shown. Lastly, we show absorbed plasma proteins such as albumin and other molecules, which are important components of GCX. **b** This electron micrograph shows a left ventricular myocardial capillary explanted from a rat and stained with Alcian blue 8GX [50]. Bar = 1 μm. **c**, **d** We used RF/FS TEM to visualize a cultured EC GCX of several micron thickness on rat fat pad ECs, shown in **c**, and bovine aortic ECs, shown in **d** [58]. Bar = 2 μm applies to both **c** and **d**. Heparan sulfate (HS) GAG, chondroitin sulfate (CS) GAG, hyaluronic acid (HA) GAG, syndecan (SDC) core protein, glypican (GPC) core protein, Glycosylphosphatidylinositol (GPI)

In favorable blood flow conditions, endothelium protection and functionality are governed by expression of the GCX [15, 19, 32, 43–51] that acts as both a buffer and a force transmitter. The GCX is a negatively charged, heavily hydrated, polysaccharide mesh layer that coats ECs [3, 50]. When GCX is intact, its pores block infiltration of large blood components while permitting absorption of plasma proteins such as 7-nm-sized albumin (Table 1) as well as smaller solutes [52–54]. The plasma proteins contribute to the thickness of the GCX (Table 1) [54–56]. In the end, GCX thickness is 0.02 to 8.9 μm in vivo (Fig. 2), and due to preservation artifacts it is usually thinner in vitro (0 to 3 μm). We were the first to use rapid freezing/freeze substitution transmission electron microscopy (RF/FS TEM) in vitro to visualize a cultured EC GCX of several micron thickness (Fig. 2) [58].

**Table 1:** This table summarizes the most widely known GCX constituents and their function

Major GCX constituent families	Well-known family members	Functions
Glycosaminoglycans (GAGs) and Sialoglycoproteins	Heparan Sulfate	GCX function is determined based on concentration and organization of GAGs.
	Chondroitin Sulfate	GCX thickness and protrusion into the vascular lumen is derived from the lengthy (hundreds to thousands) disaccharide units that make up the GAGs.
	Hyaluronic Acid	
	Sialic Acid	The strong negative charges carried by the disaccharide units further extend the GCX.
Proteoglycans	Glypicans Syndecans	These are backbone molecules that have attachment sites for tethering the GAGs. Proteoglycan family members play an important role in incorporating the extracellular GCX into the EC body. Glypicans are glycosylphosphatidylinositol anchored to the caveolae compartment of the cell membrane. Syndecans are transmembrane and connected to cytoskeleton.
Glycoproteins	Selectins	Glycoproteins reside near the GCX base and are adhesive when exposed.
	Integrins	E-Selectin and P-Selectin contribute to EC interactions with cells in the blood circulation, i.e. leukocytes and platelets.
	Immunoglobulin Superfamily	Integrins control interaction between ECs and surrounding extracellular matrix (i.e., collagen, fibronectin) as well as neighboring cells.
		Immunoglobulins act as ligands for integrins on leukocytes and platelets and contribute as mediators of adhesion to the endothelium.
Plasma Proteins	Albumin	Plasma proteins penetrate GCX pores (≤ 7 nm, when GCX is intact) and prevent GCX collapse.
		Albumin transports spingosine-1-phosphate (S1P), which binds to S1P receptors and, as a result, inactivates matrix degradation enzymes and subsequently protects against GCX shedding.

GCX is connected to the ECs via its several glycoprotein and proteoglycan backbone molecules [20]. The glycoproteins, which are at the base of the GCX (and typically buried by other GCX components (Fig. 2)), are protein-glycan conjugates (Table 1) [54]. They are adhesion molecules that can contribute to shifting physiological conditions to a pathological state when overactive [44]. The three families of adhesive molecules that are important to the GCX structure include the selectin family, the integrin family, and immunoglobulin superfamily (Fig. 2 and Table 1) [20, 54]. Selectins, predominantly found in the endothelium include E-selectin and P-selectin, both contributing to leukocyte-EC interactions (Fig. 2 and Table 1) [54, 59]. Integrins control the interaction of platelets with the apical EC surface (Fig. 2 and Table 1) and attach to collagen, fibronectin, and laminin in the subendothelium (Table 1) [44, 54]. The immunoglobulins include intercellular adhesion molecules 1 and 2, vascular cell adhesion molecule 1, and platelet endothelial cell adhesion molecule 1 [20]. They act as ligands for integrins on leukocytes and platelets and participate as crucial mediators of adhesion to the endothelium (Fig. 2 and Table 1) [20, 44, 54].

GCX proteoglycans are given more attention than the glycoproteins, due to the role that proteoglycan core proteins play in incorporating the extracellular GCX into the EC body (Table 1) [51, 54]. Glypican core proteins are glycosylphosphatidylinositol anchored to the caveolae compartment of the cell membrane (Fig. 2 and Table 1) [20, 54]. Syndecan core proteins are transmembrane and connected to the cytoskeleton (Fig. 2 and Table 1) [20, 54]. The core proteins covalently bond glycosaminoglycan (GAG) chains and sialoglycoproteins [51], including heparan sulfate, chondroitin sulfate, hyaluronic acid, and sialic acid (Fig. 2 and Table 1) [20, 44, 54]. These structures extend into the extracellular space [54]. The GAGs are lengthy due to their hundreds to thousands of disaccharide units [51]. In addition, they carry strong, negative charges that create inter-GAG repulsion forces [51]. GAG length and negative charge both significantly contribute to the extension of the GCX into the vascular lumen [51].

GCX integrity is dependent on blood chemistry and flow patterns along the walls of the vasculature. When blood chemistry includes the heavy presence of various hormones, neurotransmitters, and vasoactive factors, the GCX can be degraded to expose its glycoprotein components that facilitate thrombosis and inflammation [21]. The GCX is most notably impacted by blood flow patterns. Along vessel walls exposed to uniform blood and in relatively good health, the GCX is robust and thus protects the endothelium [60]. In geometrically complicated vessels where portions of the vessel wall are exposed to non-uniform flow patterns and predisposed to atherogenesis, GCX thinning has been noted to occur on the resident ECs [2, 4, 5]. Van den Berg et al. [5] determined that the endothelial GCX thickness at the sinus region of a mouse internal carotid artery, located at the arterial bifurcation, was significantly less than the thickness of the GCX layer located on the endothelium lining the common carotid artery. They also concluded that impaired barrier properties of GCX contribute to the enhanced LDL accumulation in the intima at the carotid artery bifurcation of mice [61]. These results provide evidence suggesting that flow patterns and associated shear stresses contribute to varying GCX dimensions, modifying GCX ability to protect the endothelium and guard against atherosclerosis [8, 19, 20].

## Pro-Atherogenic Endothelial Dysfunction as a Result of Glycocalyx Loss

In this section, we briefly report original and current findings obtained by our research group. These findings contribute to the body of knowledge about the role of the GCX in EC-dependent atherosclerosis onset and progression, summarized above.

### Increased Trans-Endothelial Permeability

It has been demonstrated in recent studies [2, 46, 62] and others that endothelial GCX helps to reinforce the barrier between the blood and underlying vascular tissue, filtering molecules and cells from crossing this barrier. Our research group is currently studying the impact of GCX loss on impairment of the barrier in vitro [32] and in vivo [2].

In vitro, we have examined cultured rat ECs that naturally express robust GCX. We have confirmed that, as expected, the intact GCX has a pore size that accepts 7 nm albumin but excludes 10 nm particles coated with neutrally charged polyethylene glycol [32]. We modified the biochemical environment of the cultured ECs by applying heparan sulfate-specific GCX cleavage enzymes or via reduction of protein content in the culture media [32]. Degradation enzyme thinned the GCX by 33% and reduced heparan sulfate content by 59% [32]. Protein content reduction led to 31% thinner GCX, with heparan sulfate content shrinking by 34% [32]. In both modified biochemical environment cases, degraded GCX permitted EC uptake of six- to sevenfold more 10 nm particles than the low level of uptake observed in healthy EC with intact GCX. This study confirms the importance of the GCX in regulating uptake of small molecules [32].

In other in vitro experiments, we assessed the impact of GCX integrity on EC uptake of LDL, which range in size from 60 to 80 nm [63]. Rather than manipulating the GCX through biochemical intervention, we modified it via the fluid mechanics environment surrounding the cultured ECs. Specifically, rat ECs were conditioned by 6 h of laminar flow. To create a physiological model, a region of a single monolayer of ECs was in direct contact with a non-uniform flow pattern, which translates to spatially variable shear stresses. An adjacent region of the same monolayer was directly exposed to a uniform flow pattern of 15 dyne/cm^2^ shear stress. In uniform shear stress conditions, the cells expressed their most abundant and continuous GCX, and oxidized LDL uptake was low. Non-uniform shear stress conditions impacted specific subcomponents of the GCX differentially. We found that non-uniform flow decreased the thickness of the heparan sulfate of the GCX by 11%, and we observed a 48% reduction in the extent to which heparan sulfate covered ECs. For the sialic acid component of the GCX, on the other hand, non-uniform flow resulted in a 21% decrease in thickness and a 44% decrease in coverage. These disturbed flow-induced changes to the GCX components correlated to heterogeneity in cellular uptake of oxidized LDLs. Oxidized LDL can be defined as a particle derived from circulating LDL that may include peroxides or their degradation products generated within the LDL molecule or elsewhere in the body associated with the particle [64]. LDL can be oxidized by vascular endothelial cells, smooth muscle cells or macrophages [65]. Oxidized LDL binds to its lectin-like receptor LOX-1 in endothelial cells which in turn triggers the CD40/CD40L signaling pathways, which then synthesizes chemokines and cell adhesion molecules [66–69]. Class B scavenger receptor CD36 mediates macrophage uptake and degradation of oxidized LDL, which transforms them into foam cells [70]. Many cells per monolayer, not all of them, internalized oxidized LDL in the cytoplasmic compartments. A lack of GCX was apparent in a majority of the cells that contained cytoplasmic oxidized LDL. These results provide evidence to support the notion that disturbed flow-induced GCX degradation is an initiator of pro-atherosclerotic EC behavior [Harding et al., unpublished results, and manuscript in preparation].

To complement in vitro studies, we demonstrated GCX integrity implications for barrier function and molecular permeability in vivo. This study examined atherosclerotic animals, which were young C57BL/6 J–background apolipoprotein-E deficient mice on a high-fat diet, rich in saturated fats and cholesterol, for 10 weeks [2]. The diet leads to significantly high levels of total cholesterol, including LDL and cholesterol elevation, which were observed in the blood circulation of the mice [2]. However, lipid was only retained in the blood vessel walls exposed to disturbed flow conditions, such as the lower curvature of the aortic arch and the outer wall of the brachiocephalic artery branch. GCX covering the plaque-filled wall was found to be discontinuous, spanning only 71% of the wall surface at a thickness of 0.85 μm [2]. Lipids were excluded from the blood vessel walls exposed to uniform flow, including the common carotid arteries and the descending thoracic aorta. In these non-plaque regions, GCX covered 97% of the blood vessel wall and had a thickness of 1.2 μm [2]. This work reinforces the hypothesis that GCX shedding correlates to blood vessel wall retention of lipids and other molecules, propagating atherosclerosis.

### Altered Inter-Endothelial Communication

The role of the GCX in regulating inter-endothelial communication is understudied. We find these junctions to be of great interest because they are complex and mediate a number of vasculoprotective EC functions [36–38]. In the previous work, it was noted that the transmembrane syndecan core proteins of the GCX are connected to the EC cytoskeleton, which interacts with intercellular junctions through zona occludin 1 (ZO-1) [71]. ZO-1 is important for gap junction docking at the cell membrane [72–74]. Subsequently, our research group has been one of the few to assess GCX function by probing its control of communicating gap junctions in cultured rat ECs.

We first established a baseline integrity of the GCX to ensure a healthy GCX layer was present. We then either enzymatically degraded the heparan sulfate component of the GCX, or repaired the GCX by allowing the cells to self-recover the heparan sulfate. Baseline and repaired GCX coverage of EC monolayers were statistically similar. Enzymatically degraded GCX covered 35% less of the endothelium. We correlated the relative GCX coverage to expression of the proteins that make up communicating gap junctions, called connexins. We also confirmed the extent to which connexin expression enabled communication activity of the gap junctions, by counting the number of gap junction-coupled cells. With baseline GCX, 60% of EC borders expressed connexin isotype 43 (Cx43), and individual cells could communicate to neighboring cells through a line of up to three cells. Heparan sulfate degradation decreased Cx43 expression to 30% and impaired the ability of individual cells to communicate. This is the first evidence that GCX effectively regulates the level of open communication between ECs [Mensah et al., full manuscript has been submitted for publication and is in review].

Human ECs are of interest for our communication studies because human ECs have been shown to represent a more realistic in vitro model for studying human vascular tissue health and disease [75]. We recently started to map the human cultured EC GCX and communication with experimental flow conditions that replicate the flow patterns that are characteristic of the atherosclerosis-resistant and atherogenic blood vessel regions. To date, we have characterized the sialic acid component of the GCX and showed that ECs express a substantial amount of sialic acid when exposed to anti-atherosclerotic uniform flow conditions of 15 dynes/cm^2^ shear stress. Pro-atherosclerotic non-uniform flow conditions lead to 54% reduction in the EC surface covered by sialic acid. At the same time, sialic acid thickness is reduced by 60% in non-uniform flow conditions [these are unpublished results from Mensah et al. The manuscript is currently in preparation].

We hypothesized that heightened GCX expression induced by uniform flow correlates to elevated communication between neighboring human ECs, based on the earlier EC studies that we conducted using rat cells. We postulated that degraded GCX caused by non-uniform flow attenuates human EC communication. Unexpectedly, we learned that the overall level of human EC-to-EC gap junctional communication is high regardless of the flow conditions [76]. These results were clarified when we separated the communication into connexin-specific components. We showed for the first time in vitro that flow is correlated to the type of connexins underlying gap junctional communication in cultured human EC monolayers [76]. In uniform flow (lesion-resistant) locations, which are home to GCX-rich ECs, Cx43 function is low while connexin isotype 40 is the dominant communicator [76]. Cx43 only plays a major role in communication in non-uniform flow regions, where GCX-deficient ECs reside [76]. These results confirm that connexin protein specificity of gap junctional communication is determined by flow pattern [76], which has a synergistic effect with GCX structure.

Collectively, these studies imply that biochemistry and fluid mechanics of blood environment determine connexin protein participation in gap junctional communication, via GCX restructuring. The connexin isoforms lead to different gap junction properties, adapting the inter-cytoplasmic flux of ions, metabolites, and other small molecules [77] that regulate endothelium phenotype relevant to vascular health as well as atherosclerosis.

### Deregulation of Nitric Oxide

Nitric oxide plays several anti-atherosclerotic roles—i.e., inhibition of platelet aggregation, prohibition of excessive smooth muscle cell proliferation, prevention of leukocyte adhesion, and vessel dilation capabilities [31]—as previously mentioned. Therefore, impairment of EC production of nitric oxide can be detrimental at every stage of atherosclerosis development. Impairment of nitric oxide can occur by a number of mechanisms. One major mechanism involves a decrease in nitric oxide production as a consequence of reduced eNOS expression, eNOS inactivation, or dislocation of active eNOS from the caveolae rafts that are located in the apical EC membrane [78]. Another mechanism involves diminished nitric oxide bioavailability via the action of nicotinamide adenine dinucleotide phosphate (NADPH) oxidases, which contribute to oxidative stress [79]. The balance between nitric oxide production by eNOS and inhibition of its bioavailability by NADPH oxidases determines the ability of nitric oxide to bring about vasodilation.

We previously elucidated the contribution of the GCX to shear stress regulation of the activity of eNOS, an enzyme that synthesizes nitric oxide [80]. In brief, in bovine ECs we confirmed that loss of the heparan sulfate component of the GCX abolished conversion of uniform flow into eNOS activation [70]. We probed deeper and found that the heparan sulfate-bound glypican-1 core protein is a key player in the mechanism by which heparan sulfate transduces uniform flow-derived forces into eNOS activity [70]. Silencing glypican-1 blocked flow-induced eNOS activation [70]. This result can be explained by the fact that glypican is normally anchored to and functional in the caveolae that contains eNOS and other signaling molecules [51]. Glypican-1 removal and its discontinued interaction with eNOS apparently interferes with the process by which eNOS is activated.

Expanding upon the previously published work, experiments are ongoing to determine the differential effects of uniform laminar shear stress versus non-uniform laminar shear stress on GCX involvement in flow-regulated eNOS activation. These experiments, once again, involve exposing monolayers of rat ECs to 6 h of side-by-side uniform and non-uniform flow. As mentioned above, the GCX is abundant and continuous in uniform flow conditions, while impaired in non-uniform flow. Specifically, the heparan sulfate component of the GCX is 11% thinner and spans 48% less endothelium surface area. The sialic acid component is 21% thinner and covers 44% less endothelium. This GCX impairment reduced eNOS activation by 50% in non-uniform flow as compared to uniform flow. We also examined caveolin-1, a protein that is a main component of caveolae. We confirmed caveolin-1 and eNOS co-localization in uniform flow and, for the first time, showed that non-uniform flow disrupts 49% of the co-localization in a GCX dependent manner. These experiments, taken together, imply that in blood vessel regions with atherogenic disturbed flow conditions, loss of GCX results in eNOS deactivation and eNOS-caveolae separation. These outcomes show that GCX degradation will have detrimental consequences for regulation of vascular tone [Harding et al., unpublished results, and manuscript in preparation].

To date, there has been no report of studies clarifying whether the GCX plays a role in EC control of nitric oxide bioavailability in response to flow. In a previous study, upon exposure to uniform flow ECs isolated from different human subjects expressed similar levels of eNOS and nitric oxide [81]. However, compared to ECs from subjects who were more at risk for cardiovascular disease, ECs from subjects with lower risk exhibited a lesser amount of flow-induced NADPH oxidase 4 (NOX4) [81]. This previous work implies that in disturbed flow conditions, NOX4 expression would be significantly higher than in uniform flow settings. Elevated NOX4 will diminish nitric oxide bioavailability, an accepted marker of endothelial dysfunction [23]. Future studies are needed to clarify if there exists GCX-mediated mechanisms by which EC under various flow stimuli differentially regulate nitric oxide bioavailability via NOX.

## Can Atherosclerosis Treatment Be Improved by Targeting Glycocalyx?

### Standard-of-Care Atherosclerosis Treatments

Numerous commercially available standard-of-care treatments are utilized to reduce atherosclerosis and cardiovascular events such as myocardial infarction, stroke, and transient ischemia attacks [82, 83]. They are largely focused on alleviation of hyperlipidemia and thrombotic complications [82].

Statins, the most well-known atherosclerosis treatment, lower lipid levels in the blood and help stabilize atherosclerotic plaques [83]. Crisby et al. and others have shown the significant benefits of pravastatin use [84]. Following the treatment period, atherosclerotic plaques were surgically removed from both treated and untreated (control) patient groups for histological analysis. The analysis showed that plaques removed from patients who were given pravastatin had significantly less lipid content and, consequently, less inflammatory cell infiltration [84]. Additional beneficial effects of pravastatin treatment included reduction in matrix degradation enzymes [84], which were correlated with higher collagen content in pravastatin-treated plaques [84]. Another side effect was inhibition of cell death [84]. Reduced matrix degradation and lower cell death, taken together, fend off formation of necrotic cavities and large voids in atherosclerotic lesions. This led Crisby to conclude that pravastatin-treated patients have more stable atherosclerotic plaques [84]. The implication is that pravastatin reduces risk of plaque rupture, which is the most common cause of strokes and myocardial infarctions [2].

Aspirin is another well-known anti-atherosclerotic drug. Aspirin has multiple effects, including anti-inflammation and reduction of thrombus formation [83]. Aspirin’s primary mechanism of action involves platelet deactivation, reducing platelets’ ability to release the bioactive substances that promote platelet aggregation and thrombus formation [82]. Platelet activity is a physiological requirement to control bleeding and is not considered effective for primary prevention of atherosclerosis [82]. Aspirin-induced platelet inhibition has been shown to be effective only as a secondary atherosclerosis preventative measure for patients who exhibit more advanced stages of the disease. In a case-control analysis, the combination of aspirin with the aforementioned statin proved to yield significant reduction in mortality [83]. It has also been shown that aspirin’s effect can be enhanced by co-administration with, clopidrogel, prasugrel, or ticagrelor, agents that block platelet surface receptors as a means of inhibiting platelet aggregation [82, 83].

### Emerging Glycocalyx-Targeted Therapies

A very promising new approach is to develop therapies that will promote GCX health in order to reverse endothelial dysfunction, an early hallmark for atherosclerosis. With stabilized GCX structure and function, we expect that ECs will block lipoprotein deposits and macrophage uptake in the blood vessel wall, arresting progression of early plaque. Additionally, GCX therapy could potentially stimulate anti-atherosclerotic EC cell-to-cell communication and nitric oxide signaling pathways. GCX-targeted therapy would enable early atherosclerosis treatment. Unfortunately, there are currently no drug therapies on the market that are approved for the purpose of restoring or protecting the GCX. Also, there are few commercially available drugs for which the benefits to GCX are known, beyond the officially approved indications. Emerging GCX-targeted approaches include stabilizing its structure, replacing it with substitutes, blocking GCX degradation enzymes, and enhancing GCX synthesis. A comprehensive summary of these approaches is described in a recent review paper written by Tarbell and Cancel [51]. Our lab has explored the replacement and structural stabilization approaches briefly described below.

Albumin has long been known to stabilize the GCX, and albumin deficiency has been known to lead to GCX collapse [85]. Clinically, it has been used to restore hemostasis after trauma [86]. However, the impact of albumin treatment on GCX has been overlooked clinically, until recent cell culture and pre-clinical studies of spingosine-1-phosphate (S1P), which is transported by albumin [87, 88]. S1P has been found to modulate the structure of the GCX by binding to S1P receptors, which has the effect of inactivating matrix degradation enzymes and subsequently protecting against GCX shedding [60, 89]. This allows de novo synthesis of GCX to outweigh its shedding, stabilizing the GCX structure [60, 89].

Success with GAG replacement has been reported in cell culture models, pre-clinically, and in studies conducted in human subjects. Our group successfully repaired the GCX with exogenous heparan sulfate and rendered the EC impermeable to small particles (10 nm in size); counteracting the effect of GCX degradation enzymes to which the ECs were also exposed [90]. Other groups have rebuilt the GCX using commercial sulodexide, which contains a combination of heparin (80%) and dermatan sulfate (20%). It has also been demonstrated that sulodexide use leads to restoration of endothelium barrier function, inhibition of GCX degradation enzymes, restraint of inflammatory activity, and deceleration of EC age-related deterioration and programmed cell death [91, 92]. A recent study demonstrated that treating obese atherosclerotic mice with chondroitin sulfate inhibited the expression of pro-inflammatory cytokines, decreased the number of monocytes migrating to inflamed cells, and reduced macrophage presence in arterial plaques [93]. Rhamnan sulfate, a non-animal polysaccharide that mimics heparan sulfate, has been successfully used in other studies on blood vessel ECs to prevent and treat conditions associated with transendothelial permeability, including inflammation and atherosclerosis [94–97]. These GAG replacement strategies are all promising approaches to prevent atherosclerosis and related cardiovascular diseases.

Our lab has recently found it necessary to co-administer a GCX structural stabilization agent, S1P, with a GCX replacement compound, exogenous heparan sulfate, in order to repair GCX in a manner that reverses highly complicated endothelial dysfunction, impaired gap junctional communication. We found that stabilizing GCX structure alone did not lead to recruitment of sufficient gap junction proteins (connexins) to the EC cell borders, limiting gap junction formation and inter-cytoplasmic communication. GCX component replacement, alone, aided in gap junction protein (connexin) expression and docking to EC cell borders but did not result in open lines of communication between neighboring ECs. Ultimately, GCX repair by treating cells with the exogenous GCX component and the GCX stabilizing co-factor restored gap junction protein placement, which translated to the reactivation of gap junction channel activity. The results of this study are encouraging. [Mensah et al., full manuscript has been submitted for publication, and a provisional patent application entitled “GlycoFix (Structurally and Functionally Repaired Endothelial Glycocalyx)” was filed in July 2017]. Pre-clinical studies in animals are now underway to test the efficacy and plausibility of heparan sulfate and S1P as a preventative and therapeutic measure to combat atherosclerosis.

## Conclusions

The endothelium forms an essential component of the vasculature and is crucial for atheroprotection because of its structural barrier function and its biological activities. The latter includes actions of endothelial cell-derived vasoactive factors such as vasodilators, via suppression of smooth muscle cell growth, and by inhibition of inflammatory responses, among a number of other functions [21, 98] (Table 2). Endothelial dysfunction can lead to a disruption in vascular homeostasis causing arterial wall damage and contributing to early stages of atherosclerosis. Proper functionality of the endothelium is highly dependent on the condition and expression of its GCX, which contributes to barrier functionality, cell-to-cell communication, and vascular tone regulation (Table 2). Our knowledge of GCX structure and function could potentially open new avenues for preventing and treating atherosclerosis.

**Table 2:** This table summarizes the role of the GCX in endothelium function, in blood vessel health or disease, and as a potential therapeutic target

Atherosclerosis	• Most common precursor to cardiovascular diseases such as strokes and myocardial infarctions • Initiated by excessive accumulation of LDLs in luminal region of blood vessel walls • Disturbed flow regions of vessel bifurcations atheroprone, with degraded or compromised GCX in these regions
GCX structure and location	• Negatively charged heterogeneous polysaccharide that lines the luminal wall of blood vessels • Primarily consists of heparan sulfate, hyaluronic acid, sialic acid, and chondroitin sulfate
GCX-mediated endothelium functions	
Barrier Function	• GCX acts as a barrier between the blood and vessel walls, filtering small molecules, lipoproteins, and circulating blood cells that seek to permeate vessel walls • A degraded or collapsed GCX has been shown to increase permeability of molecules and inflammatory cells • Healthy GCX reduces permeability
Cell-to-cell communication	• GCX attached to endothelial cell cytoskeleton which has a link to communicating gap junctions • Degraded GCX showed a decrease in gap junction protein (connexin) expression as well as communication activity
Vascular tone	• GCX has a role in the production of vasodilatory factor nitric oxide • eNOS, the enzyme that produces nitric oxide, can be significantly decreased by non-uniform flow and/or when GCX is also degraded
Standard atherosclerosis treatment options	• Statins (lipid lowering therapy) • Aspirin (anti-platelet therapy)
GCX as therapeutic	• Strengthening the GCX to counteract its degradation can restore barrier function, cell-to-cell communication, and vascular tone • Viable preventative and treatment option for addressing atherosclerosis

## Abbreviations

GCX: Glycocalyx
EC: Endothelial cell
CVD: Cardiovascular disease
ZO- 1: Zona occludin 1
Cx43: Connexin isotype 43
eNOS: Endothelial nitric oxide synthase
EDHF: Endothelium derived hyperpolarizing factor
GAG: Glycosaminoglycan
S1P: Sphingosine-1-phosphate
LDL: Low-density lipoprotein
RF/FS TEM: Rapid freezing/freeze substitution Transmission electron microscopy
NADPH: Nicotinamide adenine dinucleotide phosphate
NOX4: NADPH oxidase 4
